# Medical and Philosophical Intelligence

**Published:** 1816-08

**Authors:** 


### MEDICAL AND PHILOSOPHICAL INTELLIGENCE.

*ROYAL SOCIETY.*—May 2, Dr. NIXON communicated an account, by Dr. Serres, of a singular case of complete aphony cured by electricity. The subject was a young French officer, who, at the battle of Dresden, was in the act of giving the word of command, when a ball passed him, the effect of which on the air knocked him down, at the same time rendering him speechless, and for a day almost insensible. Two men near him were killed by the ball, which did not touch him. In the hospital at Dresden he partially recovered the use of his left side and hearing, which were impaired; but but all efforts to recover his voice were in vain, and he was discharged as an invalid. His hearing was still very confused, but his smell was preternaturally acute, and the smell of coffee was altogether intolerable to him. His tongue had contracted into a small protuberance in his mouth about the compass of an inch, and his left side continued benumbed, till he was prevailed on to be electrified by Mr. Tinman of Brussels. He had not been electrified above seven or eight times when his hearing was improved, and his tongue began to expand. Mr. T. then passed shocks through his mouth and down to his stomach, when he hastily got up, and in a low voice returned thanks to the operator, and ran off to Amsterdam like a person deranged. He returned, however, in a few days perfectly cured in his voice, which is now better than it was before the accident; but he still feels some pain in his left leg, and occasionally in foggy weather an oppression of the chest. After eight months and twenty-five days he recovered his voice completely.

On Thursday, May 23, a paper, by Sir Everard Home, on the Formation of Fat in the Tadpoles and Frogs, was read. An anatomical description of the tadpole of the rana paradoxica was given, and likewise a set of chemical observations on the eggs of birds and the spawn of frogs, by Mr. Hatchett. The yolk of birds' eggs is a mixture of albumen and a buteraceous oil. But the spawn of the frog contains no oil whatever. Hence Sir Everard concludes the necessity of tadpoles to form a quantity of fat before they can complete their change into a frog. And, as the intestines of the tadpoles are longer than those of any other animal, Sir E. considers it as evident that this fat is formed by the intestines.

On Thursday, May 30, a paper, on the Application of Galvanism to the Cure of certain Nervous Diseases, by Dr. Wilson Philip, was read. The author, in the papers already submitted to the Society, conceives that he has shown a radical difference between the nervous and the sensorial energies. Galvanism, if it be not the very same with the former, may be substituted for it, and acts in the same way. He thinks, therefore, that galvanism can be applied successfully only to disorders of the nervous system; though it may, at times, perhaps, in consequence of the intimate connection between the sensorial and nervous systems, successfully excite the former to activity. Asthma appears to be entirely a disease of the nerves; for the lungs, even in obstinate chronic cases, are not in the least injured. He therefore thought of applying galvanism as a cure of that disease, and found it of material benefit in all the cases tried. It was had recourse to in eighteen cases in the Worcester Infirmary, and in four cases of private practice. In every one of them immediate benefit was experienced; in most of them it afforded greater relief than any preceding medicine tried, and in two of the cases it produced a complete cure. The method was to apply a piece of tin foil to the nape of the neck, and another to the pit of the stomach. The wires from the two ends of a galvanic battery were connected with these, and the galvanism was continued for about ten minutes. At first it was very weak, being confined to three or four pairs of plates of four inches square, excited by water mixed with $\frac{1}{30}$th of its weight of muriatic acid; but was gradually increased till it consisted of 20 or 25 pairs of plates, by removing one of the wires along the battery. The galvanism was applied once in 24 hours; and in two cases, in which it produced permanently beneficial effects, it was applied twice in 24 hours.

June 13. T. A. Knight, Esq. F.R.S. in a letter to the President, communicated the results of some further experiments which he has made on the leaves of plants, tending to prove that the matter of timber is formed in them, and that the true sap, descending from the leaves to the roots, forms alburnum. He made a transverse and longitudinal section of the leaf-stalk of a vine, and raising up the bark an inch below the leaf, inserted it in the section of the leaf stalk; and thus preserving a communication between the bark and leaf, the intermediate part being rolled tight with paper, he found that the bark acquired thickness, and a woody character. He next took the leaf of a potato and planted it; but, although it did not, as he expected, produce tuberous roots, it formed, like cuttings of shrubs previous to their striking root, a large round lump, which lived through the winter, and he is now trying whether it will grow into a perfect potato. He took a shoot of a vine detached from the stock, and immersing a part of its largest leaf in water during a month, the smaller leaf not only lived but grew, and acquired thickness: this he considers a clear proof that the smaller leaf was nourished and augmented only by the food it received from the large one, which was partly in water. In addition to these experiments, Mr. Knight observes, that if trees be deprived of their leaves their fruit never ripens; that evergreens bear fruit at all seasons of the year, in winter as well as summer; and that deciduous plants only produce their fruit previous to shedding their leaves. The common holly is seen with its berries in the midst of winter. The quality and quantity of fruit, he seems to think, almost entirely depend on the nature and quality of the leaves; and hence the necessity of gardeners being more cautious in stripping off the leaves of fruit-trees where they tend to keep the sun off the fruit.

The following remarks of the ingenious Dr. Brewster will, we trust, prove one of the means of directing physiologists to divest themselves more and more of the mechanical powers and the properties of inorganic matter, when inquiring into the phenomena observed in living bodies.—June 20. Dr. Brewster, in a letter to the President, gave an account of his experiments on the eyes of fishes, by which it appears that the crystalline lens of fishes, according to its density, is capable of receiving the property of double refracting crystals, the same as glass, by compression. Dr. B. related a number of experiments which he made on the eyes of fishes, and their effects on colours; from which he concludes that one part of a fish's eye experiences mechanical dilatation, and another mechanical nical contraction. He thinks (and we perfectly agree with him) that the chief cause why so little progress has been made in augmenting our knowledge of the eye and of vision, is an excessive confidence in a supposed analogy between optic glasses and the crystalline lens.

---

*Linnean Society.*—Friday, May 24. On this day the Anniversary Meeting of the Linnean Society of London was held at the Society's house in Gerard-street, Soho, for the election of a Council and Officers for the ensuing year, when the following Members were declared to be of the Council, viz.

Sir James Edward Smith, Knt. M. D.
Samuel, Lord Bishop of Carlisle
Edward Forster, Esq.
George Bellas Greenough, Esq.
William Kent, Esq.
Aylmer Bourke Lambert, Esq.
William Horton Lloyd, Esq.
William George Maton, M.D.
Daniel Moore, Esq.
Rev. Thomas Rackett.
Joseph Sabine, Esq.
John, Lord Bishop of Salisbury.
Edward, Lord Stanley.
Thomas Thomson, Esq.

And the following were declared to be the Officers for the present year, viz.

Sir James Edward Smith, Knt. M.D. President.
Samuel, Lord Bishop of Carlisle, }
Aylmer Bourke Lambert, Esq. } Vice-Presidents.
Edward, Lord Stanley, }
William George Maton, M.D. }
Edward Forster, Esq. Treasurer.
Alexander Mac Leay, Esq. } Secretaries.
Mr. Richard Taylor, }

The Members of the Society afterwards dined together at the Freemasons' Tavern, Great Queen-street, according to annual custom.

June 4. A. B. Lambert, Esq. Vice-President, in the chair. Read part of a Monograph of the British Roses, by Joseph Woods, Esq. F.L.S.

June 18. W. G. Maton, M.D. Vice-President, in the chair. Read a part of "Observations on the Linnæan Junci growing in Great Britain," by J. E. Bicheno, Esq. F.L.S.

Adjourned to the 5th of November.

---

The following Report is interesting, not only on account of the source from which it is issued, but as containing a communication from his Majesty Henry (Christophe), King of Hayti.

*Report of the National Vaccine Establishment, for the Year* 1815; *dated* 31*st May*, 1816.

*To the Right Honourable Lord Viscount* Sidmouth, *Principal Secretary of State for the Home Department, &c. &c. &c.*

My Lord,—Within the last year the surgeons of our different stations in London have vaccinated 6,581 persons, and have distributed tributed to the public 32,821 charges of vaccine lymph. We cannot state precisely what the sixty-eight honorary and corresponding vaccinators may have effected in the country, as returns are not always sent: however, we have ascertained that those practitioners whom we have supplied with lymph have vaccinated 42,667 in the course of the year.

We have the satisfaction of informing your lordship, that we have furnished the means of disseminating this blessing in the island of St. Domingo; and that the director has received the annexed letter from the government of Hayti on that subject.

It is equally gratifying to us to state, that, by the ingenuity of Mr. Giraud, of Faversham, means have been devised of preserving the lymph in a fluid state; by which we have just reason to hope that it may be found efficient in any climate, and for any space of time.

Your lordship has probably been informed, that in consequence of the decisive measures adopted in Russia, Sweden, Germany, France, and Italy, the small-pox has become a very rare disease in those countries; and that, by like means, it is no longer known in Ceylon and at the Cape of Good Hope. It is a source of sincere regret to us, that it should not be equally so in this kingdom; and still more so, as this is not attributable to the casual occurrence of that disease, but, we believe, entirely to the practice of inoculation, which seems to be adhered to on interested or mistaken motives.

In Edinburgh, Glasgow, and Norwich, inoculation is disused; and, in consequence, the small-pox is scarcely known. In the country about Aberystwith in Wales, and Bawtry in Yorkshire, it has entirely disappeared. The reverse is found unhappily to be the case in Portsmouth, Bristol, and London. In the metropolis alone, the mortality by small-pox may be estimated at a thousand annually: perhaps throughout the United Kingdom it is not less than ten times that number.

We beg to conclude by stating, that it appears to us, this waste of human life can be prevented only by such legislative enactments as will entirely put a stop to the inoculation for the small-pox.

The Board is happy in stating, that it has no occasion to ask Parliament this year for any sum of money beyond that usually granted.

(Signed) J. Latham (President of the Royal College of Physicians), President.

*Henry Cline*, Master of the Royal Col. of Surg.

*Henry Halford*, M.D.; *William Lambe*, M.D.; *Joseph Agar*, M.D.; *J. Coxe*, M.D. } Censors of the Royal College of Physicians.

*William Norris*, *James Earle*, } Governors of the Royal College of Surgeons.

By order of the Board,
*James Hervey*, M.D. Registrar.

Palace

Palace of Sans Souci, Feb. 5, 1816.
13th Year of our Independence.

*The King of Hayti to Mr. James Moore, Director of the British National Vaccine Establishment, &c. &c.*

Sir,—Mr. Prince Sanders has presented me with the work which you sent me on the small-pox: I have accepted this work with pleasure, and thank you infinitely for your honourable and obliging attention, and the interest which you evince for the Haytians.

The precious discovery of vaccination is too important to human life, and does too much honour to humanity, not to induce me to adopt it in my kingdom. On the arrival of Mr. Prince Sanders, I put vaccination in use with a view to make it generally followed by the Haytian practitioners;—we have an innumerable quantity of children to vaccinate.

It is my intention to give every possible latitude to the happy results of this immortal discovery, which I had not hitherto been able to put in practice, in consequence of the disappointment which I met with in the applications I made at Jamaica, St. Thomas, and in the United States of America, relative to this object, the salutary effects of which I am well acquainted with. This benefit will still add to the gratitude of the Haytians for the great and magnanimous British nation.

I have charged Mr. Prince Sanders to testify to you personally my sincere thanks. (Signed) Henri.

---

It is more than half a century since the surgeon Garangeot pretended that he had seen a nose which had been bit off in a quarrel, thrown on the ground, and allowed to get cold again, fixed to the face, and adhering as at first. This did not at first excite even surprise; but the miracle was at length called in question, the narrator was generally ridiculed, and no one attempted to repeat the pretended operation. However, a fact, no less extraordinary, which took place in Scotland, has been judicially attested. A finger, entirely cut off, adhered again in a few days, losing merely the nail. It seems, even from different authors of the 16th century, that a lost nose was sometimes supplied by flesh taken from the arm.

M. Percy, who might have had more opportunities than any man of practising these animal engraftments, and who tried them upon dogs, whose sores are so easily cured, could never succeed. He has seen members adhere again, or pieces of flesh, which were only attached by a single tack; but this tack has always been to him a necessary condition. He does not pretend, however, that others may not be more fortunate; on the contrary, he requests surgeons to try every means to render common an operation which seems at first sight to contradict every thing that we know of the animal economy in the higher classes of animals.—*Annals of Philos.*

---

*Lightening.*—A flock of sheep, (180 in number,) the property of Mr. Roskelly, of Ringworthy Farm, on the borders of Dartmoor, were left in a field all well, on Monday evening, the 8th instant, and early on the following morning, sixty-two of them were found lying dead, killed by lightening; their eyes were forced from the sockets, and their bodies appeared as in a state of putrefaction.

We think it right to record the above, as a description of the effect of death, induced by lightening. The same happened about thirty years ago, when a lamb was killed by an experiment, made before the Royal Society, in the morning. It was intended by some of the gentlemen to partake, on the same day, of a dinner, of which the subject of the experiment was to make a part; but, before the dinner hour, putrefaction had so completely taken place, as to frustrate the wishes even of the least delicate.

We would remark further, that lightening or electricity, is among those means of sudden death, which Mr. J. Hunter considered as inducing absolute universal death, in which the muscles never contract, the blood never coagulates, and the whole runs immediately into putrefaction.—*Times Newspaper.*

---

*To the Editors of the London Medical and Physical Journal.*

GENTLEMEN,

THE knowledge of Medical Botany being a task imposed with great propriety on all students in medicine, previously to their being qualified for passing the examination at Apothecaries' Hall, and the apparent difficulties with which this study has hitherto been attended, have induced me to give you the following short sketch of the school I have formed, for the purpose of enabling persons to acquire a knowledge of this science with comparative facility.

I have composed a little Pocket Compendium of Botany, in which is given a concise introduction to the Linnæan classification, and the technical terms used in the science, with the generic and specific descriptions of all our British plants, set up in tables agreeably to the plan some time since adopted by Graffer and Galpine, in their Compendiums of the British Flora, in which the plants are numbered and put into alphabetical order. This comprises one small pocket volume, which is intended for use in the fields, when we are on our excursions, which has enabled me to conduct in a mode different from what I have hitherto been able to accomplish, by saving much time and inconvenience to the student.

The second volume contains the descriptions of the virtues and uses of each vegetable, and is thus divided:—1st. Plants useful in agriculture, including the grasses, grains, artificial grasses, &c. in which their properties, culture, quantities of seed used, and the soil to which each is best adapted. 2dly. Plants useful in rural economy, including all those that afford utensils for domestic purposes, from the oak-tree to the rush of which our candle-wicks are formed. 3dly. Plants useful in medicine, in which all their medical properties are explained, distinguishing those of the London Pharmacopœia from such that have, from time to time, been discarded by the College, together with all such as are used for culinary purposes; and a dissertation on preparing and drying herbs of all kinds; this concludes with a particular description of the poisonous vegetables, their best recommended antidotes, and also all such as are noxious, either to cattle, or as weeds, with the best mode of extirpating each.

Being prepared with a work of this description, the student requires little more than to have a systematic arrangement of the characters of each plant explained, as they are found growing. Thus, in our investigations, we have an opportunity of observing the growth, and the changes which each plant assumes, and, when in bloom, we collect specimens of each; after which, we meet and give a lecture more particularly on the distinguishing characters and properties of each; these are afterwards put into an Hortus Siccus, and prepared for a future reference.

Thus, I have lived to accomplish what I believe will be found the only method of teaching with facility this necessary and useful science to all descriptions of persons; and have now the heartfelt satisfaction of finding those gentlemen who have honoured me with their attendance for the last and present season, competent to pass any examination, as to their knowledge of the characters, names, and uses to which most of our plants, growing wild or cultivated near London, are adapted.

Confident, therefore, of the utility of my plan, and flattered with my success, and the encouragement I have received, I hope to continue every spring to give a course of twelve lectures and excursions, to conclude in July; and then to commence a summer course, which will end in September, so as to afford persons an opportunity of attending, when in London, or of obtaining any botanical information of which they may stand in need. And, for the particular purpose of affording an opportunity of having all the plants used in medicine under one view, I have lately planted these on a large scale—a quarter in my botanic garden, for the purpose of more easy reference thereto. I am, most respectfully,

Gentlemen, yours, &c.

*Botanic Garden, Sloane-street;* W. Salisbury.
*July* 25, 1816.

Mr. Salisbury's *Botanical Excursions, July* 1816.

June 28th.—Battersea Fields and gardens in that neighbourhood.

Scabiosa columbaria.

Galium erectum.

——— anglicum.

Plantago media.

Centunculus minimus.

Alchemilla vulgaris.

Sagina cerastoides.

Lysimachia thyrsiflora.

Samolus Valerandi.

Valeriana rubra (in garden).

Polycarpon tetraphyllum.

Parietaria officinalis.

Viscum album.

Anchusa sempervirens.

Scandix cerefolium.

Carum Carui.

Œgopodium Podagraria.

Statice Armeria (gardens).

Myosurus minimus.

Leucojum æstivum.

 Acorus

Acorus Calamus.
Festuca pratensis.
Avena elatior.
Festuca fluitans.
Rumex crispus.
Œnantha crocata.
Rumex obtusifolius.
Scabiosa arvensis.
——— nigra.
Avena flavescens.
Vicia Cracca.
Festuca elatior.
Iris Pseudacorus.
Stachys palustris.
Carduus palustris.
Cynosurus cristatus.
Leontodon hispidum.
Butomus umbellatus.
Sparganium ramosum.
Lapsana communis.
Crepis tectorum.
Polygonum aviculare.
Vicia sativa.
Centaurea Cyanus.
Scirpus palustris.
Aria cœspitosa.
Triglochin palustre.
Juncus effusus.
——— glaucus.
Alisma plantago.
Carex Pseudo Cyperus.
Veronica anagallis.
Bromus squarrosus.
Potentilla anserina.
Juncus conglomeratus.
Convolvulus arvensis.
Chara tomentosa.
——— hispida.
——— flexilis.
Zannichellia aquatica.
Veronica spicata (gardens).
——— scutellata.
Orchis bifolia.
Ophrys ovata.
——— apifera.
Iris fœtida.
Nardus stricta.
Berberis vulgaris.
Sedum anglicum.
Cratægus torminalis.
Thlaspi campestre.

July 8th.—Kensington Gardens and Bayswater.

Agrostis setacea.
Fagus Castanea.
Holcus canatus.
Phalaris arundinacea.
Aira aquatica.
Rumex palustris.
Polygonum amphibium.
Potamogeton gramineum.
Ceratophyllum submersum.
Sisymbrium Nasturtium.
Veronica officinalis.
Achillea millefolium.
Sagina apetala.
Peplis Portula.
Potamogeton natans.
Sison inundatum.
Agrostis fascicularis.
Festuca ovina.
Poa cristata.
Chenopodium Bonus Henricus.
Cuscuta europœa.
Arenaria rubra.
Geranium pusillum.
Rosa canina.
Potentilla reptans.
Plantago major.
Anagallis arvensis.
Sisymbrium amphibium.
Ligustrum vulgare.
Fumaria lutea.
Hordeum pratense.
Alopecurus agrestis.
Echium vulgare.
Hieracium dubium.
Daucus Carota.
Hypericum quadrangulare.

July 12th.—Battersea Fields.

Tilia europœa.
Prunella vulgaris.
Lychens Flos Cuculi.
Papaver Rhœas.
Erysimum officinale.
Plantago lanceolata.
Geranium dissectum.
Crepis biennis.
Hordeum pratense.
Urtica dioica.
Phleum pratense.
Sambucus nigra.
Dactylis glomerata.
Galium Aparine.
Senecio Jacobœa.
Phleum nodosum.
Alopecurus pratensis.
Solanum Dulcamara.
Rhinanthus Crista Galli.
Festuca Loliacea.

Rubus fruticosus.

Lolium perenne.

Bromus racemosus.

Fumaria officinalis.

Trifolium pratense.

Œnanthe fistulosa.

Lathyrus pratensis.

Lamium album.

Euphorbia helioscopia.

Bryonia alba.

Myosotis scorpioides.

July 20th.—Battersea Fields.

Artemisia vulgaris.
Chenopodium album.
Polygonum Hydropiper.
Hydrochæris Morsus Ranæ.
Hyoseris minima.
Agrostemma Githago.
Peucedanum Silaus.
Lemna trisulca.
Lemna minor.
Scutellaria galericulata.
Polygonum Convolvulus.
Circœa lutetiana.
Malva rotundifolia.
Mercurialis annua.
Sison amomum.

July 23d.—Croydon, West Wickham, and Shirley Common.

Melica cœrulea.
Lycopodium inundatum.
Conyza squarrosa.
Senecio erucifolius.
Hypericum pulchrum.
——— humifusum.
——— elodes.
Anagallis tenella.
Scutellaria minor.
Euphrasia officinalis.
Agrimonia Eupatorium.
Cistus Helianthemum.
Serapias grandiflora.
Campanula hybrida.
Papaver Argemone.
——— dubium.
Geranium columbinum.
Gnaphalium germanicum.
Juncus bufonius.
Ranunculus hederaceus.
Spergula arvensis.
Gnaphalium rectum.
Aira flexuosa.
Erica vulgaris.
Radiola millegrana.
Sagina apetala.
Hyoseris minima.
Erica cinerea.
Drosera rotundifolia.
Carex distans.
Scirpus acicularis.
Ophrys ovata.
Hieracium sylvaticum.
Chironia Centaurium.
Matricaria Chamomilla.
Hieracium prenanthoides.
Campanula rotundifolia.
Œnothera biennis.
Gnaphalium sylvaticum.
Festuca decumbens.
Malva moschata.
Spergula pentandra.
Carex stellulata.
Viola palustris.
Hydrocotyle vulgaris.
Cuscuta Epithymum.
Aira canescens.
Erigeron purpureum.
Antirrhinum Linaria.
Sonchus arvensis.
Lithospermum arvense.
Galeopsis tetrahit.

The *Excursions for the following month* will be made every Saturday; to meet at the Botanic Garden, at ten o'clock. The season has been so very unfavourable, that our Summer Course will not commence till the first week in August. The lectures at the Garden continue to be read on Mondays, at three.

---

*Middlesex Hospital.*—Dr. P. M. Latham and Dr. Southey will begin their Lectures on the Practice of Physic in the first week of October, at the Hospital.

---

Dr. Squire and Dr. Davis will commence a Course of Lectures on the Theory and Practice of Midwifery and the Diseases of Women and Children, on Tuesday, the 6th of August.

REPORT

### REPORT OF DISEASES.

The diseases of the present month have not materially varied from the last. Inflammatory complaints can scarcely be said to be less severe, but they are much less frequent; indeed, the subjects which have fallen a victim to them, or that have been reduced by the necessary discipline, may sufficiently account for the decreased numbers.

Scarlatina is very frequent, and shows itself in its various forms, but, for the most part, all of them mild, though often ushered in by very violent and somewhat anomalous symptoms. One case commenced with all the threatening aspect of phrenitis, afterwards of enteritis. Before the reporter saw the case, the throat had swelled to be a considerable degree, with the characteristic sloughs, yet was entirely unnoticed by the patient, on account of his previous sufferings. Small-pox and chicken-pox continue, the former for the most part mild, though the bills of mortality mention several deaths. These we conceive must be among the children of extreme poverty, if the above registers can be at all depended upon. Some cases *post vaccinationem*, have occurred in the usually mitigated form.

A case, somewhat new to the reporter, was presented very lately. A well-grown boy, between nine and ten years old, was observed to grow more delicate in his figure for some months. By what accident, it does not appear; the mother discovered that his testicles had almost disappeared. On examination, it was difficult to find them, and, when discovered, they did not seem larger than two full-grown pease. This suggested a doubt whether they might not have been in the abdomen at the time of birth; in which case Mr. Hunter first remarked, and, it is now well known, that they rarely, if ever, acquire their natural figure. But the mother asserted, that he was born with them of the usual size, and that they continued to grow for some time afterwards. As he had formerly been troubled with a cutaneous complaint, it was, at one time suspected, that the decrease might have been the effect of a beginning of that kind of elephantiasis, which occurred some time since in St. Bartholomew's, and is described in several late numbers of our Journal. At present, there are no marks of disease of any kind about the boy. Have any of our readers met with a similar case, without previous inflammation in those organs, or in so young a subject?

Meteoro-

### METEOROLOGICAL REGISTER.

*From June the 26th, to July the 25th,* 1816.

Kept by C. BLUNT, Philosophical Instrument Maker, No. 38, Tavistock-Street, Covent-Garden.

| Moon. | Day. | Wind. | Barometrical Pressure. | | | Temperature. | | | |
|---|---|---|---|---|---|---|---|---|---|
| | | | Max. | Min. | Mean. | Max. | Min. | Mean. | |
| | 26 | SE | 29·79 | 29·79 | 29·79 | 65 | 45 | 55· | Fair |
| | 27 | E | 29·81 | 29·50 | 29·60 | 64 | 46 | 55· | Fair |
| | 28 | E | 30·05 | 30·05 | 30·05 | 64 | 47 | 55·1 | Fair |
| | 29 | SE | 30·13 | 30·13 | 30·13 | 63 | 48 | 55·1 | Rain |
| | 30 | SE | 30·20 | 30· | 30·10 | 64 | 47 | 55·1 | Fair |
| | 1 | E | 29·75 | 29·55 | 29·65 | 63 | 45 | 54· | Rain |
| | 2 | NE | 29·82 | 29·80 | 29·81 | 63 | 43 | 53· | Rain |
| ◐ | 3 | NE | 29·86 | 29·82 | 29·84 | 61 | 43 | 52· | Rain |
| | 4 | NE | 29·88 | 29·77 | 29·83 | 62 | 41 | 51·5 | Rain |
| | 5 | E | 29·83 | 29·72 | 29·78 | 61 | 39 | 55· | Rain |
| | 6 | NE | 29·88 | 29·88 | 29·88 | 60 | 40 | 50· | Rain |
| | 7 | NW | 29·88 | 29·84 | 29·86 | 61 | 41 | 51· | Rain |
| | 8 | W | 29·70 | 29·70 | 29·70 | 62 | 41 | 51·5 | Rain |
| | 9 | W | 29·68 | 29·64 | 29·66 | 63 | 42 | 52·5 | Rain |
| ○ | 10 | NW | 29·58 | 29·58 | 29·58 | 60 | 40 | 50· | Rain |
| | 11 | N | 29·60 | 29·60 | 29·60 | 60 | 40 | 50· | Rain |
| | 12 | N | 29·80 | 29·74 | 29·77 | 61 | 40 | 50·5 | Rain |
| | 13 | NW | 29·95 | 29·95 | 29·95 | 63 | 41 | 52· | Rain |
| | 14 | NW | 29·80 | 29·80 | 29·80 | 61 | 42 | 51·5 | Rain |
| | 15 | W | 29·64 | 29·66 | 29.67 | 60 | 52 | 51· | Rain |
| | 16 | W | 29·69 | 29·67 | 29·68 | 61 | 43 | 52· | Rain |
| ◑ | 17 | W | 29·63 | 29·60 | 29·62 | 63 | 45 | 54· | Rain |
| | 18 | SW | 29·47 | 29·39 | 29·43 | 63 | 51 | 57· | Rain |
| | 19 | SW | 29·57 | 29·46 | 29·52 | 64 | 52 | 58· | Rain |
| | 20 | S | 29·75 | 29·75 | 29·75 | 80 | 55 | 67·5 | Fair |
| | 21 | SW | 29·76 | 29·76 | 29·76 | 65 | 44 | 54·5 | Rain |
| | 22 | SW | 29·77 | 29·77 | 29·77 | 65 | 45 | 55· | Rain |
| | 23 | SW | 29·59 | 29·58 | 29·58 | 68 | 46 | 57· | Rain |
| ● | 24 | SW | 29·58 | 29·56 | 29·57 | 67 | 47 | 57· | Rain |
| | 25 | W | 29·60 | 29·58 | 29·59 | 69 | 48 | 58·5 | Rain |

RESULTS.

Mean barometrical pressure of the month 29·82
Maximum 30·16, wind at W
Minimum 29·47, ——— SW

Mean temperature of the month 53·7 deg.
Maximum 80, wind at S
Minimum 42, ——— SW

*Scale exhibiting the prevailing Winds during the Month.*

| N | NE | E | SE | S | SW | W | NW |
|---|---|---|---|---|---|---|---|
| 2 | 4 | 4 | 3 | 1 | 6 | 6 | 4 |

| | Mean barometrical pressure. | Mean temperature. |
|---|---|---|
| From the new moon on the 25th June, to the first quarter, on the 3d July | 29·87 | 54·3 |
| ——— the first quarter on the 3d, to the full moon on the 9th | 29·78 | 51·9 |
| ——— the full moon on the 9th, to the last quarter on the 17th | 29·71 | 51·37 |
| ——— the last quarter on the 17th, to the new moon on the 24th | 29·62 | 58 |

